# Valorization of *Rapana venosa* Viscera Through Enzymatic Production of Protein Hydrolysates

**DOI:** 10.1155/sci5/8589143

**Published:** 2026-05-30

**Authors:** Bilge Bilgin Fıçıcılar, Koray Korkmaz

**Affiliations:** ^1^ Fatsa Marine Sciences Faculty, Department of Fisheries Technologies Engineering, Ordu University, Ordu, Turkey, odu.edu.tr

**Keywords:** amino acids, hydrolysate, ICP-MS, LC-MS/MS, peptides, *Rapana venosa* waste

## Abstract

The veined rapa whelk (*Rapana venosa*) is an invasive marine gastropod with commercial value, particularly in the Black Sea region. During processing, its viscera are typically discarded as waste. This study aimed to valorize the viscera by producing protein hydrolysates through enzymatic hydrolysis and lyophilization. Proximate analysis showed that the viscera contained 22.61% protein, 2.87% fat, and 2.95% ash. The hydrolysates were analyzed for amino acids using LC‐MS/MS and minerals via ICP‐MS. Glutamic acid, aspartic acid, and phenylalanine were the most abundant amino acids, with essential amino acids accounting for 52.4% of the total. The mineral profile revealed high levels of magnesium, calcium, and iron, along with elevated concentrations of copper and cadmium. Fatty acid analysis identified 24 components, with saturated fatty acids (SFAs) being the most abundant group, followed by polyunsaturated fatty acids (PUFAs) and monounsaturated fatty acids (MUFAs). Major fatty acids included palmitic acid (C16:0, 13.69%), oleic acid (C18:1n‐9, 3.21%), eicosapentaenoic acid (EPA, 4.56%), and docosahexaenoic acid (DHA, 8.15%). EPA and DHA together made up 12.71% of total fatty acids, highlighting the nutritional potential of the viscera. These findings support the potential use of *Rapana venosa* viscera hydrolysates in nutraceutical and functional food applications, although further investigation into heavy metal content is required.

## 1. Introduction

The veined rapa whelk, *Rapana venosa*, is a marine gastropod mollusk belonging to the family Muricidae, which is native to the western Pacific, including the Sea of Japan, the Yellow Sea, East China Sea, and the Bohai Sea. However, this species has become invasive in many parts of the world, such as the Black Sea, the Adriatic and Aegean Seas, and the Chesapeake Bay [1, 2]. As a successful invader, *Rapana venosa* spread to Bulgaria, Turkey, and Romania between 1959 and 1972. The introduction *of Rapana venosa* to these regions is believed to have occurred via the transportation of oysters from the Sea of Japan for aquaculture enhancement efforts. This species preys on mussels and has a spawning period from June to early August [1, 3, 4].

*Rapana venosa* holds significant commercial value for all Black Sea countries. These species are primarily harvested using dredges and diving methods along the Turkish Black Sea coast. Although not consumed domestically within Turkey primarily due to the absence of a cultural tradition of gastropod consumption and limited local market awareness, it is exported to international markets [5].

*Rapana venosa* is exported to numerous countries, primarily Japan, South Korea, Taiwan, and the People’s Republic of China, as a frozen product processed in factories. In 2025, Türkiye exported approximately 1943 tons of *Rapana venosa*, generating a total revenue of approximately 20.9 million USD [6]. Considering that only 15%–20% of the total biomass corresponds to edible meat [7], while the remaining majority consists of shell and viscera, and based on an estimated total catch of 10,000–13,000 tons, approximately 8000–11,000 tons of shell waste and 500–1300 tons of viscera are generated annually, highlighting the significant potential for valorization of this by‐product.

During the processing of *Rapana venosa*, a substantial amount of the biomass, namely, the internal organs, is usually disposed away as waste. Specifically, the internal organs can contribute to a significant proportion of the overall body weight. The disposal of internal organs presents both an environmental challenge and an economic opportunity. The high protein content of these by‐products makes them ideal for conversion into protein hydrolysates, which have numerous applications in the food, nutraceutical, and feed industries. Rich in bioactive peptides, shellfish viscera offer various health benefits due to their antioxidant, anti‐inflammatory, and antithrombotic properties, presenting significant potential for therapeutic applications [8, 9].

To the best of our knowledge, this is the first study to comprehensively characterize protein hydrolysates derived from the viscera of *Rapana venos*a, a by‐product routinely discarded during commercial processing in the Black Sea region. By integrating proximate analysis, fatty acid profiling, amino acid composition (LC‐MS/MS), and elemental analysis (inductively coupled plasma mass spectrometry [ICP‐MS]), this study offers a multidimensional nutritional and biochemical assessment of an underexplored marine by‐product. These findings provide a novel scientific basis for the valorization of *R. venosa* viscera as a sustainable source of bioactive compounds for nutraceutical and functional food applications, contributing original data to the limited literature on gastropod byproduct utilization.

The main objective of this study was to chemically characterize hydrolysates obtained from the enzymatic treatment of *Rapana venosa* (sea snail) viscera, followed by lyophilization, to evaluate their potential as a value‐added ingredient for nutraceutical and functional food applications. LC‐MS/MS was employed to determine the amino acid composition of the product, while ICP‐MS was used to assess the presence of inorganic elements.

## 2. Materials and Methodology

### 2.1. Materials

*Rapana venosa* specimens were obtained from a sea snail processing facility in Fatsa, Ordu, Turkey, following a boiling process. A total of 5 kg of *Rapana venosa* samples were transported to the Seafood Processing Laboratories at the Fatsa Faculty of Marine Sciences. Each *Rapana venosa* viscera weighed approximately 12 g, resulting in the use of 417 individual samples for this study. The samples were transported to the laboratory in polystyrene containers with ice to ensure proper preservation. Upon arrival, the specimens were promptly eviscerated under laboratory conditions.

### 2.2. Proximate Analysis

The crude protein (*N* × 6.25) content of the *Rapana venosa* meat and viscera was determined using the Kjeldahl method [10]. Crude fat analysis was performed according to Bligh and Dyer [11], moisture content was determined according to [12], and crude ash content was analyzed according to AOAC [13].

### 2.3. Fatty Acid Composition

Fatty acid profiles were determined, and fatty acid methyl esters (FAME) from the extracted lipid were prepared according to the method of Ichihara et al. [14]; 25 mg of the extracted oil sample was mixed with 4 mL of 2M KOH and 2 mL of n‐heptane.

The mixture was vortexed for 2 min at room temperature and then centrifuged at 4000 rpm for 10 min, after which the n‐heptane layer was extracted for analysis using gas chromatography (GC) equipment.

#### 2.3.1. Gas Chromatographic Condition

The fatty acid composition was analyzed using a gas chromatography‐mass spectrometry (GC‐MS/FID) Shimadzu GC‐MS‐QP2010 Ultra device. The column used was a TR‐CN100 column (Teknokroma, Spain) with a length of 100 m, an inner diameter of 0.25 mm, and a film thickness of 0.20 μm. The system was equipped with a flame ionization detector (FID), and the injection was performed in split mode with a split ratio of 50.0. The injection volume was set to 1 μL, and the carrier gas (helium) flow rate was maintained at 0.94 mL/min, with a total flow of 51.1 mL/min and a column pressure of 250 kPa.

The oven temperature program began at 140°C and lasted 8 min. Subsequently, the temperature was increased by 4°C per minute to 220°C, and then by 1°C per minute to 230°C, where it was held for 15 min to complete the analysis. Fatty acids were identified by comparing the retention times of FAME with those of a standard 37‐component FAME mixture (Supelco, Catalogue No: 18919). Results were expressed as GC area percentages, representing the mean value ± standard deviation (SD) of three replicate analyses.

### 2.4. Enzymatic Hydrolysis

**FIGURE 1:**
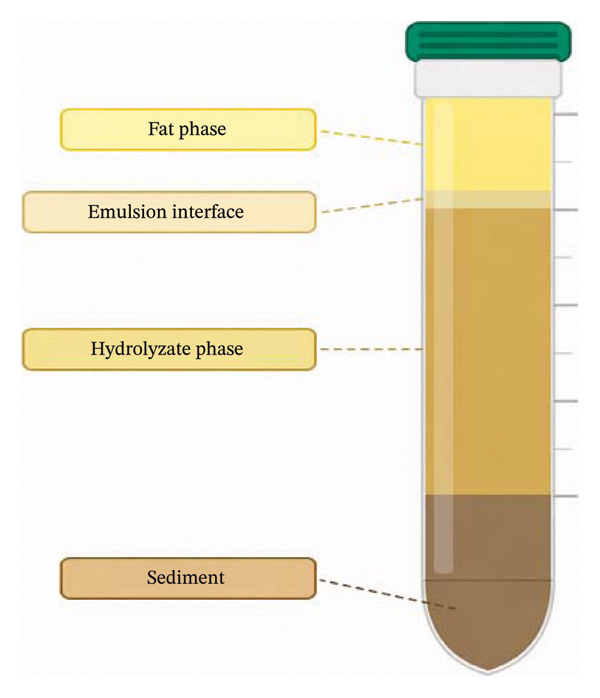
Phase separation of *Rapana venos*a viscera hydrolysate after centrifugation, showing the distinct layers of fat phase, emulsion interface, hydrolysate phase, and sediment.

Viscera of *Rapana venosa* was processed using a meat grinder (Empero, EMP.12.01.P, Turkey). The by‐products were then heated in a water bath (Memmert, WNB 22, Germany) at 90°C for 20 min to inactivate endogenous enzymes. Once cooled, the minced tissue was homogenized with distilled water at a 1:1 ratio (Ultra‐Turrax, IKA T25). Prior to hydrolysis, the pH of the medium was adjusted using 2 N NaOH to values appropriate for the optimum activity of the enzymes. Hydrolysis was carried out with Novozymes Alcalase (≥ 2.4 U/g, bacterial sourced) at an enzyme‐substrate concentration of 1% under optimal conditions (pH 8, 50°C for 60 min). During hydrolysis, the pH was monitored with a calibrated pH meter and, as no significant change was observed, no additional pH adjustment was made. Following enzymatic hydrolysis, the enzymes were inactivated according to the manufacturer’s recommendations, after which the hydrolysates were cooled at room temperature for approximately 15 min. The hydrolysate was centrifuged (Sigma 3‐30KS, Germany) at 4100 rpm for 20 min to separate into four phases: fat, light protein‐fat, protein, and insoluble material. The fat phase was removed, and the protein phase was filtered, frozen at −80°C, and lyophilized (Figure 1).

### 2.5. Total Amino Acid (TAA) and Free Amino Acid (FAA) Analysis

The TAA content in the samples was determined using a modified version of the methods employed by Lee and Hwang [15] and Chan and Matanjun [16] on an LC‐MS/MS instrument. According to this method, 0.2 g of homogenized sample was weighed into a solution containing 10 mL of 6 N HCl. After tightly sealing the mixture, the test tube was vortexed for 5 min and then placed in an oven at 110°C for 24 h to complete the hydrolysis. The mixture was then cooled to room temperature and centrifuged at 4000 rpm for 15 min at 4°C. The supernatant obtained after centrifugation was filtered through a 0.45‐μm PTFE membrane filter and injected into the LC‐MS/MS instrument for analysis.

### 2.6. Heavy Metal and Trace Element Analysis

Heavy metal and trace element analysis was performed using a Thermo iCAP RQ ICP‐MS, following the method by Intarasirisawat et al. [17]. For this, 0.1 g of sample was placed in microwave digestion vessels with 4 mL of nitric acid, sealed, and digested in a microwave system. The microwave digestion was carried out in a closed‐vessel system using nitric acid. The temperature was gradually increased to 190°C–200°C over approximately 15 min and maintained for 10–15 min to ensure complete digestion, in accordance with standard acid digestion protocols for ICP‐MS analysis. After cooling to room temperature, the digested samples were diluted prior to analysis. After digestion, the samples were transferred to volumetric flasks and diluted to 10 mL of distilled water. The diluted samples were analyzed in the ICP‐MS. Argon gas flow rates for plasma, auxiliary, and nebulizer were set at 15, 0.2, and 0.8 L/min, respectively, with a sample flow rate of 1.5 mL/min. Results were reported in mg/1000 g.

### 2.7. Statistical Analysis

Statistical analyses were performed using R software (version 4.3.3). Differences between groups were assessed by one‐way ANOVA at a significance level of *P* < 0.05. Fatty acid composition between viscera and muscle samples was compared using independent samples *t*‐test. Statistical significance was set at *P* < 0.05. Results are presented as mean ± SD (*n* = 3).

## 3. Results and Discussion

### 3.1. Proximate Composition

Proximate analyses were conducted on *Rapana venosa* viscera and meat (Table 1). The results indicated that *Rapana venosa* viscera contained 71.54 ± 0.10% moisture, 2.87 ± 0.09% fat, 2.95 ± 0.20% ash, and 22.61 ± 0.42% protein. In comparison, *Rapana venosa* meat exhibited 73.00 ± 0.95% moisture, 0.98 ± 0.31% fat, 2.07 ± 0.14% ash, and 20.12 ± 0.48% protein. These results indicate significant differences in the fat, ash, and protein contents between the viscera and meat, while the difference in water content was not statistically significant (*p > 0.05*).

**TABLE 1:** Proximate composition of *Rapana venosa* viscera and meat.

Parameter	*Rapana venosa* viscera	*Rapana venosa* meat
Water (%)	71.54 ± 0.10^a^	73.00 ± 0.95^a^
Fat (%)	2.87 ± 0.09^a^	0.98 ± 0.31^b^
Ash (%)	2.95 ± 0.20^a^	2.07 ± 0.14^b^
Protein (%)	22.61 ± 0.42^a^	20.12 ± 0.48^b^

*Note:* Values are mean ± standard deviation (*n* = 3); means in different columns with different letters are significantly different (*p* < 0.05).

After boiling, Korkmaz and Pinal [18] measured the proximate composition of *Rapana venosa* meat and reported that it contained 73.32% moisture, 2.49% ash, 19.44% crude protein, and 0.83% lipid. In a study conducted with *Rapana venosa* meat, the samples were cooked for 15 min at 105°C. The analysis revealed moisture content of 69.74% ± 0.21, protein content of 20.18%, fat content of 0.24%, and ash content of 2.29% [19]. The proximate composition of *Rapana venosa* meat is consistent across studies in terms of moisture, protein, and ash content, with only minor variations observed. However, the fat content shows a notable decrease after cooking, likely due to fat loss during thermal processing, while other components remain similar.

Several studies have found that the crude protein content in fish by‐products typically ranges from 8% to 35% [9, 20, 21]. *Rapana venosa* viscera protein content falls within this range, suggesting it has a significant protein level. Our findings indicate that the moisture content is higher than that of *Rapana venosa* meat reported by Luo et al. [22] but falls within the range reported for other marine snails, such as *Hemifusus ternatanus* [23]. The ash content in our study (2.95%) is comparable to that of other marine snails, such as *Dicathais orbita* [24]. This similarity indicates consistency in mineral content with related species. The fat content of *Rapana venosa* viscera is comparatively lower than that of certain marine snails, such as *Dicathais orbita* and *Hemifusus ternatanus* [23, 24]. However, the fat content of *Rapana venosa* viscera aligns more closely with species like *Strombus gracilior*. This suggests that the lipid profile of *Rapana venosa* viscera is relatively low, comparable to other marine snails known for their lower fat content.

### 3.2. Fatty Acid Composition of *Rapana venosa* Viscera and Meat

Fatty acids are essential constituents of lipids that carry out vital functions in biological systems, serving as energy sources, cell membrane structural elements, and precursors for signaling molecules. Polyunsaturated fatty acids (PUFAs), particularly omega‐3 (n‐3) and omega‐6 (n‐6) fatty acids, are recognized for their health advantages, including anti‐inflammatory effects and the capacity to reduce the risk of cardiovascular illnesses [25].

Table 1 presents the proximate composition of *Rapana venosa* viscera and meat. The lipid content was significantly higher in the viscera (2.87% ± 0.09) than in the meat (0.98% ± 0.31), suggesting its potential as a more abundant source of fats. The fatty acid composition was determined to assess meat and viscera’s nutritional quality and lipid profile.

**FIGURE 2:**
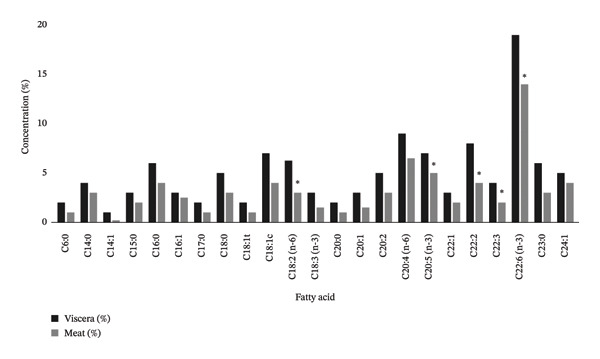
Comparison of fatty acid concentrations (%) in *Rapana venosa* viscera and meat. ^∗^indicates statistically significant differences between viscera and muscle samples (*p < 0.05*), determined by independent samples *t*‐test.

In the *Rapana venosa* samples (meat and viscera), 24 fatty acids were identified (Figure 2). In the viscera, the major fatty acids and their concentrations were C14:0 (3.02%), C16:0 (13.69%), C18:0 (6.52%), C16:1 (2.61%), C18:1t (1.10%), C18:1c (2.11%), C20:1n‐9 (13.39%), C18:2n‐6 (2.13%), C18:3n‐3 (5.31%), EPA (C20:5n‐3, 4.56%), and DHA (C22:6n‐3, 8.15%). Similarly, in the meat, the predominant fatty acids and their concentrations were C14:0 (2.04%), C16:0 (8.86%), C18:0 (6.87%), C16:1 (2.18%), C18:1c (5.18%), C20:1n‐9 (8.57%), C18:2n‐6 (3.01%), C18:3n‐3 (0.90%), EPA (C20:5n‐3, 6.06%), and DHA (C22:6n‐3, 19.26%). The significant amounts of EPA and DHA in both tissues underscore their dietary significance and potential health benefits. Notably, the DHA concentration in the meat (19.26%) was substantially higher than in the viscera (8.15%), emphasizing the meat’s superior nutritional quality regarding omega‐3 fatty acid content.

The fatty acid profiles of marine invertebrates, such as *Rapana venosa*, are shaped by environmental factors including diet, seasonal changes, salinity, and pollution [26].

In this study, saturated fatty acids (SFAs) were the most abundant group in the viscera, followed by PUFAs and monounsaturated fatty acids (MUFAs). Conversely, in the meat, PUFAs were the most abundant group, followed by SFAs and MUFAs. These results highlight the differing fatty acid compositions between the meat and viscera of *Rapana venosa*. The fatty acid composition of the meat samples aligns with findings from previous studies on Black Sea *Rapana venosa* lipids, where PUFA > SFA > MUFA was consistently reported [7, 27–29].

In the viscera samples, palmitic acid (C16:0) was the most abundant fatty acid in the SFA group, oleic acid (C18:1n9) in the MUFA group, and eicosapentaenoic acid (C20:5n3, EPA) in the PUFA group. In the meat samples, palmitic acid (C16:0) was also the most abundant FA in the SFA group, followed by oleic acid (C18:1n9) in the MUFA group and docosahexaenoic acid (C22:6n3, DHA) in the PUFA group. Marine mollusks, such as *Rapana venosa*, are distinguished by a high concentration of important n‐3 PUFAs, namely, EPA and DHA, which constitute a substantial proportion of the total fatty acids in both visceral and muscle samples [30]. In this study, EPA and DHA constituted 12.71% of the total fatty acids in the viscera, with EPA at 4.56% and DHA at 8.15%. These levels were elevated in the meat, with EPA and DHA accounting for 25.32% of the total fatty acids, with EPA at 6.06% and DHA at 19.26%.

The viscera contained lower PUFA levels than the meat, which can be explained by the distinct functional roles of these two tissues. The muscular foot is rich in structural phospholipids that selectively retain long‐chain n‐3 PUFAs like DHA to maintain membrane integrity. At the same time, the visceral mass functions primarily as a lipid storage and processing site, accumulating more SFA and MUFA‐rich triacylglycerols. Luo et al. [22] observed a similar pattern in *R. venosa*, where the visceral mass had higher total fat but a greater proportion of SFA than the muscle.

The feeding habits of *R. venosa* may also explain the distribution of fatty acids between tissues. As a bivalve predator, the dietary PUFAs it obtains are first absorbed through the visceral mass and then selectively transported to muscle tissue. This preferential routing of DHA toward muscle phospholipids has been documented in other mollusks [30] and may account for the relatively lower DHA levels we observed in the viscera. Water temperature is another factor known to shift fatty acid profiles in gastropods [26].

Similar fatty acid distribution patterns have been reported in other predatory marine gastropods. In both *Dicathais orb*it*a* [24] and *Hemifusus ternatanus* [23], SFA were the dominant lipid class in visceral tissues, while PUFA were more concentrated in muscle, consistent with our results. To our knowledge, no previous study has examined the fatty acid profile of *R. venosa* viscera in the context of hydrolysate production. Although the viscera showed lower relative PUFA content than the meat, its EPA + DHA contribution of 12.71% remains nutritionally relevant and is within the range reported for other edible marine gastropods, supporting its valorization potential.

### 3.3. Amino Acid Composition of *Rapana venosa* Viscera Hydrolysates

Protein hydrolysates, produced through enzymatic hydrolysis, contain FAAs and small peptides, typically composed of 2–20 amino acids. FAAs are generally considered more bioavailable than protein‐bound amino acids, as they do not require prior enzymatic digestion and can be directly absorbed through the intestinal epithelium [31, 32]. These provide significant benefits, such as nutraceuticals or functional foods, due to their amino acid profiles. These amino acids and peptides play crucial roles in various physiological functions, directly or indirectly contributing to overall health. The composition of these amino acids in marine‐derived hydrolysates can vary depending on the raw material, enzyme source, and hydrolysis conditions [33, 34].

The amino acid profiles of fish and shellfish proteins play a crucial role in various biological and physiological activities, contributing significantly to the maintenance of human health [35]. Studies have shown that certain amino acids, including aspartic acid, glycine, and glutamic acid, can promote wound healing [36, 37]. Hydrophobic amino acids (HAAs) can interact with membrane lipid bilayers, allowing them to reach their targets and effectively scavenge harmful radicals [38]. Amino acids with antioxidant properties, such as methionine, histidine, tyrosine, lysine, and tryptophan, have been found to have powerful radical scavenging activity in oxidative reactions [39]. The antioxidant capacity of histidine is particularly significant, as the protonation of its imidazole ring enables it to act as a highly efficient hydrogen donor, thereby playing a crucial role in strengthening the overall antioxidant defense system [40]. Additionally, amino acids with antioxidant properties can chelate metal ions, such as Fe^2+^ and Cu^2+^, effectively reducing their activity and inhibiting lipid peroxidation. Studies have demonstrated that amino acids such as glutamic and aspartic acids exhibit antiproliferative properties, suggesting their potential role in preventing and managing various types of cancer [40, 41]. In the present study, the TAA profile of *Rapana venosa* viscera revealed that 52.3% were antioxidant amino acids (AAAs), while 34.7% were HAAs (Table 2). On the other hand, the FAA profile showed that 48.9% consisted of AAAs, with 31.2% being HAAs (Table 3). These findings indicate that both the TAA and FAA compositions of *Rapana venosa* viscera are rich in AAAs and HAAs, with the TAA profile showing a slightly higher proportion of these beneficial amino acids.

**TABLE 2:** Total amino acid composition of *Rapana venosa* viscera protein hydrolysate.

Total amino acids	*Rapana venosa* viscera protein hydrolysate (mg/1000g)
*Essential amino acids (EAA)*	
Histidine	224.51 ± 0.049
Isoleucine	618.49 ± 0.028
Leucine	1081.10 ± 0.106
Lysine	2485.76 ± 0.021
Methionine	366.61 ± 0.0210
Phenylalanine	2860.39 ± 0.028
Threonine	578.09 ± 0.028
Valine	736.19 ± 0.014

*Nonessential amino acids (NEAA)*	
Alanine	341.930 ± 0.028
Arginine	362.200 ± 0.057
Aspartic acid	2414.820 ± 0.028
Glutamic acid	2626.415 ± 0.035
*Glycine*	290.420 ± 0.028
Proline	572.590 ± 0.028
Serine	377.630 ± 0.020
Tyrosine	545.605 ± 0.035
Tryptophan	77.805 ± 0.007

*Note:* Values are mean ± standard deviation (*n* = 3).

**TABLE 3:** Free amino acid composition of *Rapana venosa viscera* protein hydrolysate.

Free amino acids	*Rapana venosa* viscera protein hydrolysate (mg/1000g)
*Essential amino acids (EAA)*	
Histidine	46.62 ± 0.071
Isoleucine	171.31 ± 0.035
Leucine	196.73 ± 0.057
Lysine	243.70 ± 0.057
Methionine	79.85 ± 0.050
Phenylalanine	380.31 ± 0.141
Threonine	152.55 ± 0.035
Valine	177.31 ± 0.028

*Nonessential amino acids (NEAA)*	
Alanine	63.98 ± 0.042
Arginine	58.11 ± 0.170
Aspartic acid	224.14 ± 0.035
Glutamic acid	586.36 ± 0.035
*Glycine*	43.60 ± 0.085
Proline	140.27 ± 0.028
Serine	86.97 ± 0.010
Tyrosine	146.87 ± 0.035
Tryptophan	0.965 ± 0.007

*Note:* Values are mean ± standard deviation (*n* = 3).

The amino acid compositions of the *Rapana venosa* viscera hydrolysate and the FAAs obtained after enzymatic hydrolysis are detailed in Tables 2 and 3. In the TAA profile of the viscera, glutamic acid (2626.42 mg/1000 g) was the most abundant, accounting for a significant portion, followed by aspartic acid (2414.82 mg/1000 g) and phenylalanine (2860.39 mg/1000 g). Other notable amino acids included lysine (2485.76 mg/1000 g), leucine (1081.10 mg/1000 g), and valine (736.19 mg/1000 g). The total amount of essential amino acids (EAAs) was substantial, accounting for approximately 52.4% of the TAAs. The combined proportion of proline, leucine, alanine, and aromatic amino acids (phenylalanine and tyrosine) was approximately 41.9%.

In the FAA profile, glutamic acid (586.36 mg/1000 g) and phenylalanine (380.31 mg/1000 g) were the most prominent, although their levels were lower than those found in the TAAs. Lysine (243.70 mg/1000 g) and leucine (196.73 mg/1000 g) were also found in significant quantities. The high proportion of EAAs in the FAA pool suggests that *Rapana venosa* viscera hydrolysates could serve as an excellent source of EAAs, making them highly suitable for various nutraceutical applications. The fish protein hydrolysates and shellfish hydrolysates comprised significant amounts of glutamic acid, aspartic acid, and glycine, which are major amino acids [42, 43]. Aspartic and glutamic acids are significant amino acids found in fish and marine‐derived protein hydrolysates, crucial for protein structure. Glutamic acid is highly regarded for its ability to enhance flavor, while arginine and lysine significantly contribute to the nutritional profile, serving as EAAs. The precise concentration of these amino acids might differ based on the species of marine organism, the enzyme used in hydrolysis, and the specific conditions that regulate the hydrolysis process [44]. Compared to the literature on fish hydrolysates, the TAA and FAA content in *Rapana venosa* viscera hydrolysate was lower [45, 46]. Notably, no previous studies have specifically focused on mollusk viscera hydrolysate, which is commonly discarded as waste. Compared to fish processing by‐products, the distinct composition and structure of *Rapana venosa* viscera likely contributed to these differences in amino acid content.

The proportion of EAAs in the TAA profile of *R. venosa* viscera hydrolysate (52.4%) is noteworthy from a nutritional standpoint. This value is comparable to or exceeds that reported for fish‐derived protein hydrolysates, where EAA proportions typically range from 40% to 50% of TAAs [33, 45]. The dominance of lysine and phenylalanine among the EAAs is particularly relevant, as lysine is commonly the first limiting amino acid in cereal‐based diets, and its abundance in marine‐derived hydrolysates makes them well suited as complementary protein supplements [34]. It should be noted, however, that a complete comparison against FAO/WHO reference patterns for adults was not possible in the present study, as the amino acid analysis was performed on a targeted panel using LC‐MS/MS. Future studies employing complete acid hydrolysis profiling are recommended to enable comprehensive EAA scoring against international dietary reference standards.

### 3.4. Mineral Composition of *Rapana venosa* Viscera Protein Hydrolysate

The mineral composition of the *Rapana venosa* viscera protein hydrolysates evaluated in this study is shown in Table 4.

**TABLE 4:** Mineral composition of *Rapana venosa* viscera protein hydrolysates revealed by ICP‐MS.

Element	Concentration (Average ± SD) ppm
Aluminum	19.49 ± 3.42
Calcium	1956.49 ± 8.42
Chromium	< 0.01
Iron	81.82 ± 6.88
Magnesium	9467.916 ± 143.1
Selenium	2.98 ± 0.045
Zinc	58.87 ± 0.593

*Note:* Values are mean ± standard deviation (*n* = 3).

Magnesium (9467.916 ± 143.1 ppm) was the predominant mineral, followed by calcium (1956.49 ± 8.42 ppm), iron (81.82 ± 6.88 ppm), and zinc (58.87 ± 0.593 ppm). Selenium was also present in notable concentrations (2.98 ± 0.045 ppm). Selenium is an essential trace element known for its antioxidant properties, primarily as a component of selenoproteins such as glutathione peroxidase, which protect cells from oxidative damage. Getting enough selenium is linked to improved immune function, better thyroid hormone regulation, and a lower risk of chronic diseases associated with oxidative stress. The presence of selenium in *Rapana venosa* viscera hydrolysates further supports their potential as functional food ingredients with antioxidant benefits [47]. Compared to previous studies on fish protein hydrolysates, the magnesium, calcium, zinc, and iron levels in *Rapana venosa* viscera hydrolysates were significantly higher [45, 48]. This could be due to species‐specific differences and the use of internal organs in hydrolysis. Topcuoğlu et al. [49] reported that iron levels in the soft tissue of *Rapana venosa* ranged between 550 ± 3 ppm and 170 ± 4 ppm, while zinc levels were between 230.4 ± 0.9 ppm and 200.9 ± 0.6 ppm. Chromium concentrations were below the detection limits (< 0.06 ppm) in all samples. Bayraklı et al. (2024) analyzed boiled *Rapana venosa* meat samples and reported the following values: iron at 59.510 ppm, zinc at 20.30 ppm, chromium at 0.052 ppm, and aluminum at 46.71 ppm in boiled edible tissues.

The high mineral content, particularly magnesium and calcium, indicates that *Rapana venosa* viscera hydrolysates may have promising nutritional and functional applications. The elevated mineral levels may also be linked to the high ash content commonly observed in hydrolysates derived from mollusks.

The magnesium concentration detected in the present study (9467.9 ± 143.1 ppm on a dry weight basis) is higher than values reported for fish‐derived protein hydrolysates (822–2773 μg/g) [48]. Magnesium is an essential macromineral involved in over 300 enzymatic reactions, including energy metabolism, protein synthesis, and muscle and nerve function. It plays a particularly important role in cardiovascular health, where adequate intake has been associated with a reduced risk of hypertension, cardiac arrhythmia, and Type 2 diabetes [50]. Populations with low dietary magnesium intake, including elderly individuals, patients with gastrointestinal disorders, and those consuming low‐calorie diets, represent priority target groups that could benefit from magnesium‐enriched functional food ingredients. The adult recommended dietary allowance (RDA) for magnesium is 310–420 mg/day, and the calcium RDA is 1000–1200 mg/day for adults [51]. To contextualize these findings, the magnesium content of the present hydrolysates (9467.9 μg/g) substantially exceeds the upper range reported for salmon and mackerel backbone hydrolysates (822–2773 μg/g) [48], suggesting that the visceral origin and species‐specific mineral metabolism of *R. venosa* contribute to its elevated mineral profile. The calcium content (1956.5 μg/g) likewise exceeded the upper bound reported by de la Fuente et al. [48] (789–1786 μg/g), further supporting the enriched mineral composition of gastropod viscera‐derived hydrolysates relative to fish backbone by‐products. In terms of nutritional relevance, the adult RDA for magnesium is 310–420 mg/day, and for calcium, it is 1000–1200 mg/day [51]. While direct supplementation scenarios would require dedicated dose‐response and bioavailability studies, the high mineral density of these lyophilized hydrolysates suggests potential utility as a mineral‐enriched ingredient in functional food formulations, particularly for populations at risk of magnesium deficiency, such as the elderly, individuals with gastrointestinal malabsorption disorders, and those with inadequate dietary intake [50].

### 3.5. Heavy Metal Composition of *Rapana venosa* Viscera Protein Hydrolysate

The heavy metal concentrations of *Rapana venosa* viscera protein hydrolysates are presented in Table 5. The measured concentrations include arsenic (16.67 ± 0.300 ppm), cadmium (25.92 ± 0.428 ppm), cobalt (1.02 ± 0.017 ppm), copper (68.52 ± 0.412 ppm), manganese (2.15 ± 0.039 ppm), mercury (0.12 ± 0.041 ppm), and lead (3.08 ± 0.086 ppm). These results highlight the presence of various trace metals in the hydrolysates, with copper showing the highest concentration among the analyzed metals.

**TABLE 5:** Heavy metal composition of *Rapana venosa* viscera protein hydrolysates revealed by ICP‐MS.

Element	Concentration (average ± SD) ppm
Arsenic	16.67 ± 0.300
Cadmium	25.92 ± 0.428
Cobalt	1.02 ± 0.017
Copper	68.52 ± 0.412
Manganese	2.15 ± 0.039
Mercury	0.12 ± 0.041
Lead	3.08 ± 0.086

*Note:* Values are mean ± standard deviation (*n* = 3).

According to Commission Regulation [52] and the Turkish Food Codex [53], permissible levels for heavy metals such as lead (1.5 ppm), cadmium (1.0 ppm), and mercury (0.5 ppm) have been established for bivalve mollusks. However, these regulatory limits are specifically defined for bivalve species and do not directly apply to gastropod mollusks such as *Rapana venosa*, for which no specific maximum limits have been established.

Furthermore, these limits refer to the edible portions of mollusks, whereas protein hydrolysates may exhibit relatively higher concentrations of heavy metals than raw viscera due to the removal of water and nonprotein components during hydrolysis.

The edible part of *Rapana venosa* primarily consists of its soft tissues, specifically the muscular foot, which is widely regarded as the most consumable portion. After removing these soft tissues, the remaining nonedible internal organs are discarded. The nonedible part of *Rapana venosa* consists of the hepatopancreas (digestive gland), stomach, intestines, gills, kidneys, gonads (ovaries or testes), heart, and cerebral ganglia with nerve cords [22, 54]. The liver tissue is highly active in the uptake and storage of heavy metals, and it is well known that large amounts of metallothionein induction occur in the liver tissue of fishes. This is supported by findings from a study where heavy metal (Cd, Pb, Cu, Zn, and Fe) concentrations were measured in the muscle, gill, liver, and gonad of three fish species (*Sparus aurata, Dicentrarchus labrax*, and *Mugil cephalus*) [55].

To provide a preliminary risk assessment, the cadmium (Cd) concentration in the hydrolysates (25.92 mg/kg) was compared with the tolerable intake levels established by EFSA [56]. The tolerable weekly intake (TWI) for cadmium is 2.5 μg/kg body weight, corresponding to 175 μg/week for a 70 kg adult. Based on the measured Cd concentration, consuming approximately 6.7 g of hydrolysate would reach this weekly limit. This indicates that direct, regular consumption of the hydrolysate in its current form may pose a health risk if not properly controlled or purified. However, hydrolysates are typically used as functional ingredients at low inclusion levels rather than as bulk food products.

Although elevated heavy metal concentrations were observed in the hydrolysates, several mitigation strategies have been proposed to reduce heavy metal content in aquatic products. Among these, chelation‐based treatments are particularly effective. For example, the application of chelating agents such as sodium acetate, disodium oxalate, and trisodium citrate has been reported to significantly reduce heavy metal concentrations in mussel tissues, with removal efficiencies reaching up to 88.57% for Pb and 68.01% for Cd [57].

In addition, recent studies have demonstrated that food‐grade biopolymers and adsorbent materials, such as chitosan and its derivatives, can effectively bind heavy metal ions due to their functional groups (e.g., amino and hydroxyl groups), offering a promising and environmentally friendly approach for heavy metal removal in seafood processing systems [58]. Therefore, even though the current study emphasizes the compositional and nutritional potential of *Rapana venosa* viscera hydrolysates, further purification techniques, like chelation or adsorption‐based treatments, would be necessary before the usage in food or nutraceutical systems to guarantee safety and regulatory compliance.

## 4. Conclusion

This study analyzes the amino acid profile, fatty acid composition, and elemental content of protein hydrolysates derived from *Rapana venosa* viscera, a by‐product typically considered waste in the Black Sea region. The findings reveal that *R. venosa* viscera hydrolysates are a rich source of EAAs and nonessential amino acids, supporting their potential use as functional food ingredients. In addition, fatty acid analysis identified 24 components in the viscera, with SFAs as the most abundant group, followed by PUFA and MUFA. Notably, the viscera contained significant levels of omega‐3 fatty acids, particularly EPA (4.56%) and DHA (8.15%), which together accounted for 12.71% of the total fatty acids, highlighting their nutritional relevance and potential health benefits. However, due to the ecological characteristics of *R. venosa*, which inhabit deeper marine environments and follow feeding strategies that predispose it to the bioaccumulation of heavy metals, careful consideration must be given to potential contamination risks in future applications. This study represents a novel contribution to the literature, as it is the first to explore the potential of *R. venosa* viscera hydrolysates for functional food production, offering a sustainable pathway for valorizing marine by‐products. The environmental and economic benefits of utilizing such waste materials are promising, particularly when combined with advanced processing technologies that minimize environmental impact. Nevertheless, the present study has certain limitations that should be recognized. The enzymatic hydrolysis process was carried out on a laboratory scale, and its application to pilot or industrial‐scale production still needs to be assessed. The high heavy metal concentrations also require thorough safety evaluations before any food or nutraceutical use. Future research should focus on in vitro toxicological testing and pilot‐scale production trials to determine the scalability and safety of the process.

## Author Contributions

Bilge Bilgin Fıçıcılar: conducting experimental work, writing–original manuscript, data curation, and visualization; Koray Korkmaz: supervision and writing–original draft and reviewing and editing.

## Funding

No funds or grants were received.

## Ethics Statement

This research did not involve human subjects, live vertebrates, or any procedures requiring institutional ethical approval; hence, no ethics committee authorization was necessary.

## Consent

The authors have nothing to report.

## Conflicts of Interest

The authors declare no conflicts of interest.

## Data Availability

All data generated or analyzed during this study are included in this published article.
